# Digital dermatitis in Swedish dairy herds assessed by ELISA targeting *Treponema phagedenis* in bulk tank milk

**DOI:** 10.1186/s12917-024-04021-y

**Published:** 2024-05-02

**Authors:** Lex Roelofs, Jenny Frössling, Anna Rosander, Joakim Bjerketorp, Reza Arabi Belaghi, Ingrid Hansson, Sara Frosth

**Affiliations:** 1 Department of Animal Biosciences, Faculty of Veterinary Medicine and Animal Science, Swedish University of Agricultural Sciences (SLU), P.O. Box 7023, Uppsala, 750 07 Sweden; 2 Department of Animal Environment and Health, Swedish University of Agricultural Sciences (SLU), P.O. Box 234, Skara, 532 23 Sweden; 3 Department of Epidemiology, Surveillance and Risk Assessment, Swedish Veterinary Agency (SVA), Uppsala, 751 89 Sweden; 4 Department of Energy and Technology, Unit of Applied Statistics and Mathematics, Swedish University of Agricultural Sciences (SLU), P.O. Box 7032, Uppsala, 750 07 Sweden

**Keywords:** Lameness, *Treponema phagedenis*, Enzyme-Linked Immunosorbent Assay (ELISA), Diagnostics, Prevalence

## Abstract

**Background:**

Digital dermatitis (DD) is a contagious hoof infection affecting cattle worldwide. The disease causes lameness and a reduction in animal welfare, which ultimately leads to major decreases in milk production in dairy cattle. The disease is most likely of polymicrobial origin with *Treponema phagedenis* and other *Treponema* spp. playing a key role; however, the etiology is not fully understood. Diagnosis of the disease is based on visual assessment of the feet by trained hoof-trimmers and veterinarians, as a more reliable diagnostic method is lacking. The aim of this study was to evaluate the use of an enzyme-linked immunosorbent assay (ELISA) on bulk tank milk samples testing for the presence of *T. phagedenis* antibodies as a proxy to assess herd prevalence of DD in Swedish dairy cattle herds.

**Results:**

Bulk tank milk samples were collected in 2013 from 612 dairy herds spread across Sweden. A nationwide DD apparent prevalence of 11.9% (8.1–14.4% CI95%) was found, with the highest proportion of test-positive herds in the South Swedish regions (31.3%; 19.9–42.4% CI95%).

**Conclusions:**

This study reveals an underestimation of DD prevalence based on test results compared to hoof trimming data, highlighting the critical need for a reliable and accurate diagnostic method. Such a method is essential for disease monitoring and the development of effective control strategies. The novelty of ELISA-based diagnostic methods for DD, coupled with the disease’s polymicrobial origin, suggests an avenue for improvement. Developing an expanded ELISA, incorporating antigens from various bacterial species implicated in the disease, could enhance diagnostic accuracy. The significance of this study is underscored by the extensive analysis of a substantial sample size (612). Notably, this investigation stands as the largest assessment to date, evaluating the application of ELISA on bulk tank milk for DD diagnosis at the herd level.

## Background

Digital dermatitis (DD) is a contagious hoof infection affecting cattle that was first described by Cheli and Mortellaro in 1974 in Italy [1]. The disease is characterized by gradually worsening ulcers and inflammation on the heels of cattle [2–4]. The disease is found worldwide and is considered endemic in most countries housing cattle [5]. It is one of the major causes of lameness in cattle and is the most treated hoof disorder in the United States of America [2, 6, 7]. In Norway, the Netherlands, and Denmark, DD herd prevalence of 17%, 21%, and 85%, respectively, have been reported [8–11]. Digital dermatitis was observed visually in 55% of Swedish cattle herds in 2020, with DD lesions detected in 4.9% of all cows that underwent hoof-trimming (F. Åkerström, Växa Sverige personal communication April 20, 2022). Digital dermatitis causes reduced animal welfare with discomfort and pain in cattle, which can result in severe lameness and economic loss [12]. In severe cases, lameness and reduced mobility can last more than four months [13]. Infection with DD leads to a decrease in productivity and milk yield due to an increase in lying time and a reduction in total feeding time [12, 14, 15]. In Sweden, this decrease in productivity has been estimated to be up to 5.5 kg energy-corrected milk per day per cow [16]. For heifers that have not yet had their first calf, infection with DD can lead to an even larger decrease in milk yield during their first lactation [17]. Digital dermatitis in Swedish herds could have a large impact on milk production, and mapping national prevalence is of significant importance in the effort to prevent and control this disease and thereby improve both the welfare and productivity of cows.

The etiology of DD is not completely understood, but it is thought to have a polymicrobial origin with *Treponema phagedenis* playing a key role in infection [18–20]. *Treponema* spp*.* are the most commonly isolated bacteria from infection sites, more specifically *T. phagedenis, T. pedis* and *T. medium/ “T. vincentii”* [21–23]. *Treponema* spp*.* belong to the family *Treponemataceae* in the phylum *Spirochaetota* after recent revision by the International Committee on Systematics of Prokaryotes (ICSP), which decided that the names of phyla should be based on genera as the nomenclatural type [24]. Two well-known human pathogens belong to this family: *T.* *denticola*, a causative agent of periodontitis, and *T. pallidum*, the causative agent of syphilis [25]. These species are fastidious and slow-growing, making them difficult to culture and thereby challenging to study [26]. In addition to *Treponema* spp., other species have also been identified from infection sites, including *Mycoplasmopsis* spp., *Bacteroides* spp., *Porphyromonas* spp., *Campylobacter* spp., *Fusobacterium necrophorum, Borrelia burgdorferi,* and “*Candidatus* Amoebophilus asiaticus*”*; however, these species have mainly been identified using non-culturing methods [18–20, 27].

Currently, the diagnosis of DD is performed through visual assessment by professional hoof-trimmers [11, 28]. During these trimming sessions, hooves are inspected for irregularities, cleaned and trimmed, and if any disorders are found the cows undergo treatment [11, 28]. In 2022, a total of 2,108 dairy herds, comprising 214,000 cows were part of Växa’s ‘Kokontrollen’ program, an initiative that handles all data related to cattle of associated cattle herds in addition to acting as an advisory service in Sweden (F. Åkerström, Växa Sverige personal communication May 15, 2023). As part of ‘Kokontrollen’, a total of 534,000 hoof trimmings were recorded, and in 69.4% of the trimmings, no hoof disorders were registered (F. Åkerström, Växa Sverige personal communication May 15, 2023). These evaluations are subjective, time consuming, expensive and solely based on the abilities of the hoof-trimmer. A more accurate method to diagnose DD is therefore needed, and several promising methods are gaining ground. Several of these methods are enzyme-linked immunosorbent assays (ELISAs) that detect antibodies against *Treponema* spp. antigens, either in the form of whole-cell preparations [29–35] or as specific proteins produced using recombinant DNA techniques [36, 37]. One of these ELISAs is now being used in the Netherlands as part of a hoof health monitoring program [32]. The *T. phagedenis* PrrA antigen has been used in an ELISA with bulk tank milk samples to estimate the herd prevalence of DD with promising results [37]. Furthermore, a PrrA-based ELISA and a *Treponema* spp. whole-cell ELISA were compared against hoof-trimming data to estimate sensitivity and specificity, however both tests failed to produce acceptable sensitivity (maximum of 0.4 [0.19 – 0.86]) for the detection of DD at the herd level without compromising specificity (maximum of 0.99 [0.95 – 1.0]) [11] when Norwegian bulk tank milk samples were investigated. Although these methods are still far from perfect they are much faster and cheaper than the visual inspection of the hooves, and show potential as a screening tool for DD.

Despite the fact that DD is endemic in Sweden as well as in other European countries [2, 6–8], effective and reliable monitoring programs and knowledge on nationwide prevalence are lacking, which is key to develop effective disease prevention and control strategies. Therefore, this study aimed to investigate the use of an ELISA identifying antibodies against *T. phagedenis* in bulk tank milk to assess the apparent prevalence of DD in Swedish dairy herds.

## Methods

### Study herds

Bulk tank milk samples were collected from 612 Swedish dairy herds in all eight statistical Nomenclature of Territorial Units for Statistics level 2 (NUTS2) regions of Sweden (Table 1). The selection of herds was done in two-steps. First a random selection was made based on all milk-producing herds in Sweden (4669 at the time). This random selection was plotted on a map, and some minor changes were made manually to make sure all regions with dairy cattle in Sweden were represented. All samples were collected between April and May 2013, originally with the purpose of monitoring Schmallenberg virus infection in cattle, and stored frozen at -20 °C at the Swedish Veterinary Agency (SVA) in Uppsala, Sweden. Data analysis was done retrospectively on the collected samples. Sample size was originally calculated for the Schmallenberg study and was done using the ‘sample size to achieve specified population-level sensitivity’ by Ausvet Epitools [38], where with a perfect test specificity and a disease prevalence of 0.5%, 588 samples will give a survey sensitivity of 95%.

**Table 1:** Regional distribution of cattle herds screened for presence of *Treponema phagedenis* antibodies in bulk tank milk in Sweden

National areas	Counties within the area	Total No. of sampled herds	No. of sampled herds by region	Total number of herds in the region
**SE11: Stockholm**		**6**		**60**
	Stockholm		6	60
**SE12: East Middle Sweden**		**100**		**727**
	Uppsala		19	165
	Södermanland		22	133
	Östergötland		37	294
	Örebro		16	87
	Västmanland		6	48
**SE21: Småland and Öland**		**163**		**1301**
	Jönköping		56	454
	Kronoberg		28	190
	Kalmar		52	438
	Gotland		27	219
**SE22: South Sweden**		**84**		**490**
	Blekinge		12	71
	Skåne		72	419
**SE23: West Sweden**		**142**		**1093**
	Halland		41	265
	Västra Götaland		101	828
**SE31: North Middle Sweden**		**55**		**369**
	Värmland		13	114
	Dalarna		14	97
	Gävleborg		28	158
**SE32: Middle Norrland**		**54**		**274**
	Västernorrland		26	124
	Jämtland		26	150
**SE33: Upper Norrland**		**69**		**354**
	Västerbotten		53	250
	Norrbotten		16	104
**Total**		**612**		**4669**

### Enzyme-linked immunosorbent assay

Before sample analysis in November 2015, the samples were removed from the freezer and kept at room temperature until completely thawed. All bulk milk samples were analyzed for the presence of anti-*Treponema* antibodies using the prototype Digital Dermatitis ELISA Kit A (Medicago AB, Uppsala, Sweden).

The ELISA was based on the *T. phagedenis* protein PrrA, one of three *T. phagedenis* immunogenic proteins used in ELISA test versions evaluated in Frössling et al. [36], which found a sensitivity of 80% (CI95% 52–91%) and a specificity of 100% (CI95% 66–100%) when compared to hoof trimming records, respectively. For all wells on a coated ELISA plate containing control samples 90 μl phosphate-buffered saline containing 0.5% Tween-20 (Sigma–Aldrich, St Louis, MO, USA) (PBST) and 10 μl diluted controls were added. Bulk tank milk samples were diluted 1:2 in PBST and 100 μl was added to the remaining wells. The plates then were sealed and incubated for 2 h at 37℃. After incubation plates were washed six times with 350 μl PBST. After washing plates were inoculated with 100 μl diluted horseradish peroxidase (HRP) conjugate, sealed and incubated for 1 h at 37℃. Plates were washed again six times with 350 μl PBST. After washing plates were inoculated with 100 μl EC Blue Enhanced tetramethylbenzidine (TMB) solution. Plates were then incubated for ten minutes at room temperature away from direct sunlight, before 50 μl of stop solution was added. Lastly optical density (OD) was measured at 450 nm within 15 min of finishing the assay. The whole ELISA process was done according to manufacturer’s instructions. A percent positive (PP) value was calculated according to the formula: $\text{PP}=\frac{100\times\text{A}450(\mathrm{Test}\;\mathrm{Sample})}{\text{A}450(\mathrm{Positive}\;\mathrm{Sample})}$ where A_450_ is the mean OD value of the tested samples. All ELISA tests were performed at SVA.

A cut-off value of PP > 27 was considered positive for presence of *T. phagedenis* antibodies in bulk tank milk samples according to manufacturer’s instructions. Furthermore we looked at cut-off values for individual milk samples which were considered positive for presence of *T. phagedenis* antibodies with a PP > 20, and a cut-off value of PP > 15 which according to a study conducted by Holmøy et al. [11] increased sensitivity of the Medicago test without sacrificing on specificity.

### Statistical analysis

Statistical analyses were performed in Excel, GraphPad Prism version 9.5.1 for Windows (GraphPad Software, San Diego, CA, USA) and R Statistical Software (v4.3.0; R Core Team 2023). For calculation of 95% confidence intervals (CI95%) a normal approximation with the formula: $P\pm Z\sqrt{P(1-P)/n}$ was used, where P stands for the probability a herd was test-positive and n for the number of herds tested. The Z-value was set to 1.96 which corresponds to the Z-value for an α of 0.05 to calculate 95% confidence intervals. Chi-Squared tests for independence were performed in R.

A chi-squared test of independence was performed to see if there was a significant difference between seroprevalence of *T.* *phagedenis* antibodies in the different regions compared to the total seroprevalence.

A cluster analysis was performed to uncover hidden patterns (groups) within the population. First only the PP-value was used as a measurement for analysis without assigning labels (‘1’ for positive and ‘0’ for negative) to the classification algorithms. This was done to verify the optimal number of clusters for this dataset. Subsequently, the labeled clusters were tabulated alongside their classification as positive or negative to validate the accuracy of the classification and the specified threshold parameters. For this cluster analysis the R package NbClust was used [39]. The NbClust package provides 30 indices to determine the optimal number of clusters and suggests the best clustering scheme from the data provided by varying all combinations of number of clusters, distance measures and clustering methods.

## Results

**Fig. 1:**
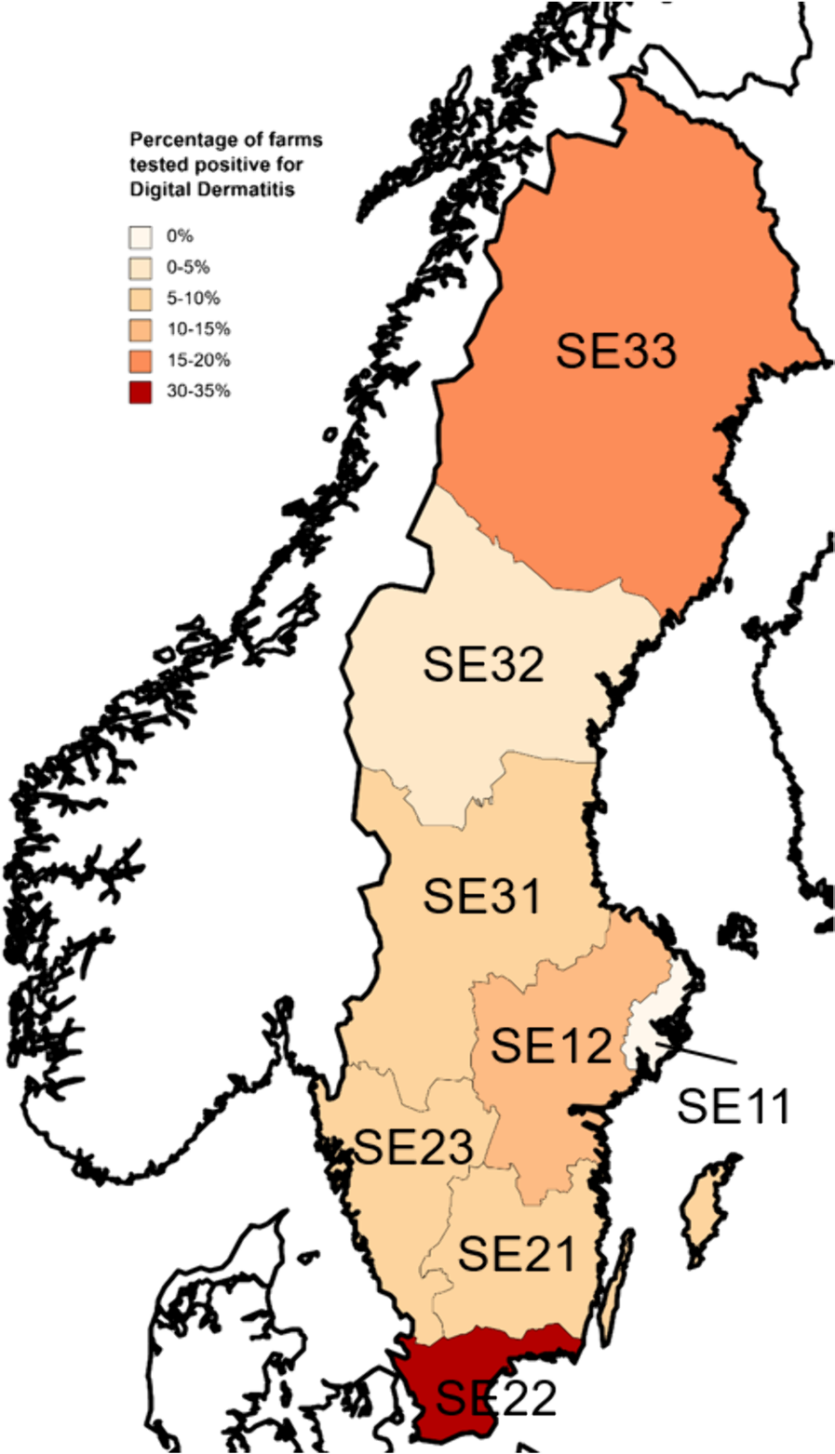
Geographical distribution of digital dermatitis in different Swedish statistical regions. Herds were classified as DD-positive based on presence of *Treponema phagedenis* antibodies in bulk tank milk

**Fig. 2:**
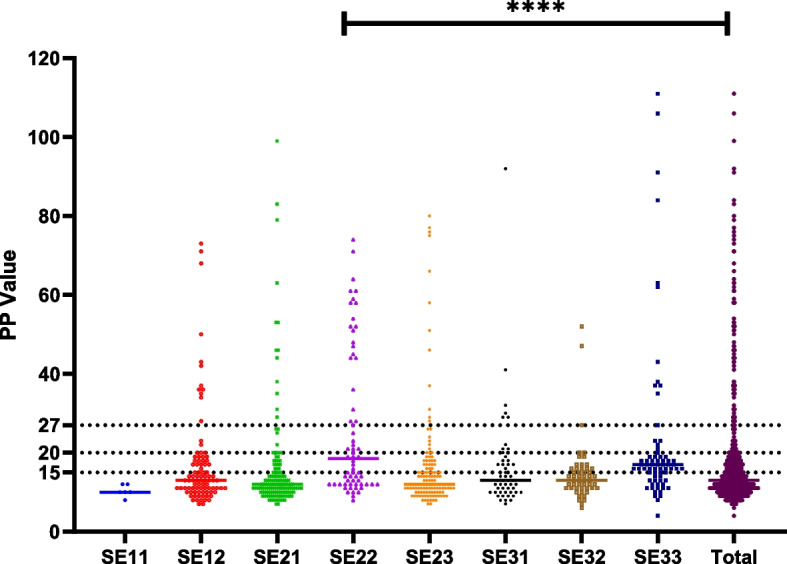
Distribution of ELISA values for presence of *Treponema phagedenis* antibodies in bulk tank milk in different Swedish statistical regions, using different percent positive cut-off values. Horizontal bars represent median values. Dotted horizontal lines represent the different percent positive cut-off values analyzed. **** *p* < 0.0005

The apparent herd seroprevalence (defined as the proportion of herds that have detectable antibody levels in bulk tank milk) for the presence of *T. phagedenis* antibodies in bulk tank milk was 73 out of 612 (11.9%; 95% CI 9.1–14.4%), as shown in Table 2. The highest herd seroprevalence was found in South Sweden, NUTS2 statistical region SE22, where samples from 20 out of 64 (31.3%; CI95% 19.9–42.6%) herds tested positive (Table 2). In contrast, the regions with the lowest number of positive tests were SE11 (Stockholm) with 0 out of 6 samples (0%) and SE32 (Middle Norrland) with 2 out of 52 milk samples (3.2%; CI95% 0–9.1%) testing positive (Table 2). A visual representation of the distribution of positive-testing herds is shown in Fig. 1. The apparent prevalence in region SE22 (South Sweden) was significantly higher than the total seroprevalence (Fig. 2) (*p* < 0.0005).

**Table 2:** Regional distribution of *Treponema phagedenis* antibodies in bulk tank milk: Proportion of positive herds across Swedish NUTS2 regions

Region	No. of herds	Median PP^1^-value	Seropositive	% Positive	CI95%
**SE11**	6	10	0	0%	NA
**SE12**	89	13	11	12.4%	5.5–19.2
**SE21**	150	12	13	8.7%	4.2–13.2
**SE22**	64	19	20	31.3%	19.9–42.6
**SE23**	130	12	12	9.2%	4.3–14.2
**SE31**	51	13	4	7.8%	0.5–15.2
**SE32**	52	13	2	3.9%	0–9.1
**SE33**	69	17	11	15.9%	7.3–24.6
***Total***	612	13	73	11.9%	8.1–14.4

^1^Percent positive

**Fig. 3:**
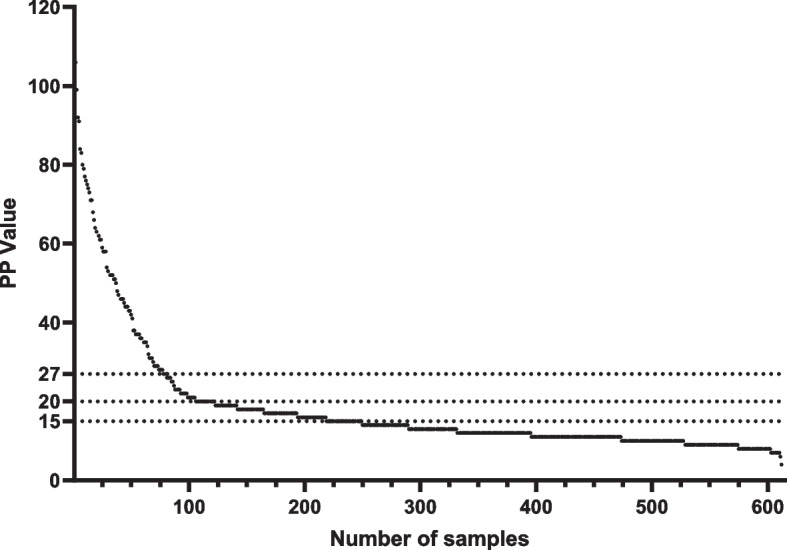
Distribution of ELISA values for presence of *Treponema phagedenis* in bulk tank milk using different percent positive cut-off values. Dotted horizontal lines represent the different percent positive cut-off values analyzed

We observed an increase in apparent seroprevalence of *T. phagedenis* antibodies in bulk tank milk for all regions when analyzing lower PP cut-off values (Figs. 2, 3 and Table 3).

**Table 3:** Apparent seroprevalence of *Treponema phagedenis* antibodies in bulk tank milk using different percent positive cut-off values

PP^a^ cut-off	Apparent seroprevalence	CI95%
> 27	11.9%	8.12 – 14.4
> 20	17.2%	8.13 – 19.6
> 15	35.7%	8.12 – 38.1

^a^Percent positive

**Fig. 4:**
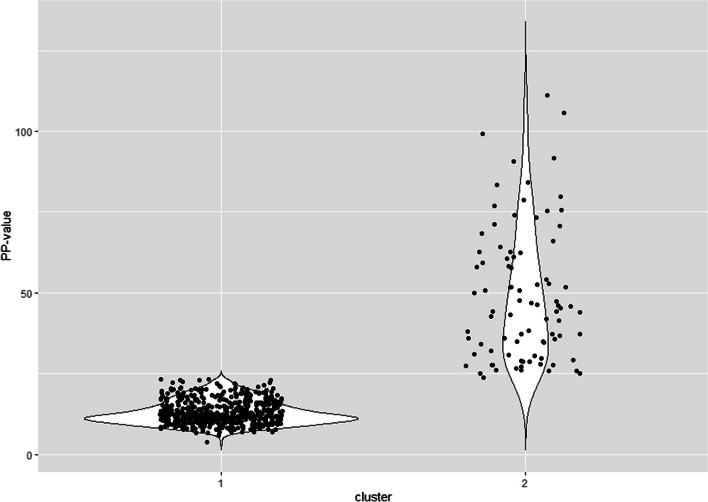
Cluster analysis showing distribution of *Treponema phagedenis* positive and negative herds based on Percent Positive value

The NbClust algorithm identified two as the optimal number of hidden groups, based on several different clustering measures. The first cluster contained 525 dairy herds (85.8%), while the second cluster included 87 dairy herds (14.2%) (Fig. 4).

## Discussion

The significance of this study is underscored by the comprehensive sample analysis, involving a total of 612 dairy herds. As the largest investigation of its kind, this study evaluates the application of ELISA for detection of *T. phagedenis* antibodies in bulk tank milk and examination of apparent prevalence of DD at the herd level [32]. Notably, while the data reflects the year 2013, it stands as the sole available dataset for Sweden, making this study a foundational baseline for future investigation into *T. phagedenis* in dairy cattle. Based on our findings, the seroprevalence of *T. phagedenis* antibodies in bulk tank milk in Swedish dairy herds in 2013 was 11.9%. Considering expected limitations in diagnostic test sensitivity [36], we anticipate that part of the true positive cases may have been incorrectly identified as negative, this is further supported by the results from our cluster analysis (Fig. 4) which showed that 14.2% of our test results cluster together instead of the 11.9% that was found. This would lead to an underestimation of the actual number of herds affected by DD in Sweden. This is further supported when looking at hoof trimming data, which suggests DD herd prevalence was as high as 38.6% in 2014 (F. Åkerström, Växa Sverige, personal communication, August 21st, 2023). Limitations in test sensitivity were also demonstrated in Holmøy et al. (2021) when the same test as in this study was used with bulk tank milk samples from Norwegian dairy herds. In the study by Holmøy et al. (2021), the authors investigated lowering the PP cut-off value to increase sensitivity of the ELISA [11]. In our study we looked at two additional cut-off values, namely 20 which is used for individual milk samples according to the manufacturer’s instructions of the kit and 15, which was shown by Holmøy et al. (2021) to be the lowest cut-off value that increased sensitivity without compromising on specificity [11]. When we analyze a PP cut-off value of > 20 we see a slight increase in seroprevalence (from 11.9% to 17.2%), however it does not get close to the reported 38.6% from hoof-trimming records. In contrast when looking at a PP cut-off value of > 15 we get much closer to the reported prevalence of DD according to hoof trimming records (35.7% compared to 38.6%). Unfortunately, due to a lack of herd specific hoof-trimming data we are unable to confirm if this estimation is due to an increase in sensitivity or an increase in false positive test results. Judging from our cluster analysis (Fig. 4) lowering the PP cut-off value for the test to 23 should be considered, as there are two distinct groups identified separated at a PP value of 23. This will slightly increase the seroprevalence of *T. phagedenis* antibodies in bulk tank milk to 14.2%.

The ELISA used only measures the presence of antibodies against the bacterium *T. phagedenis*. As the origin of the disease is polymicrobial [18–20], not including other species-specific antigens in the ELISA could be one of the reasons this suspected underestimation in DD prevalence was observed. It would therefore be of great benefit to repeat this study with an expanded ELISA containing antigens to several bacterial species involved in the infection to be able to assess trends in prevalence and geographical distribution.

Digital dermatitis was found in every statistical region in Sweden except SE11 (Stockholm); however, the number of herds tested and the number of herds in general are low in this region since it is a more urban area. The apparent prevalence of DD observed in Sweden (11.9%) was considerably lower than in Denmark (85%) but similar to that in neighboring Norway (17%), where in both cases prevalence was based on hoof trimming observations [8, 10, 11]. In Norway, a steep increase in the number of DD cases was observed from 2013 onwards; which could be attributed to the introduction of an electronic recording of hoof diseases [40]. This same phenomenon was observed in Sweden, where the proportion of infected herds increased from 38.6% to 58.5% between 2014 and 2021 (F. Åkerström, Växa Sverige, personal communication, August 21st, 2023). The increase in DD cases around Europe could partially be explained by the increase in free-stall systems, which has shown to be one of the risk factors for development of DD [41, 42]. The increase in free-stall systems is also true for Sweden. In 2020 it was reported that around 76% of Swedish dairy herds use a free stall system compared to approximately one-quarter in 2003 [43, 44].

In this study, the highest number of *T. phagadenis* antibody seropositive cases in Sweden was found in South Sweden, which includes the counties of Skåne and Blekinge. Increasing herd size has been shown to be a risk factor for the development of DD [12], as the number of cows per herd in Skåne is one of the highest in Sweden this could be a potential explanation for the steep seroprevalence found in this region [45]. In addition these counties are located near Denmark, where a very high prevalence has been documented [10]. The similarities in climate and latitude (e.g., rain, sun hours, and temperature) between the two regions could be one factor why the seroprevalence is much higher in South Sweden than in the other Swedish regions [9, 10]. There is no proof of live-animal trade between the two countries, however trade of equipment between the two regions is not ruled out. As *Treponema* spp. have been shown to survive on hoof knives under aerobic conditions, trade of equipment could potentially also play a role in the seroprevalence in this region [46].

Although the knowledge surrounding DD has increased, a reliable diagnostic method for the disease is lacking. Currently, the diagnosis of DD relies on the subjective observations of hoof-trimmers and veterinarians. Unfortunately, the early stages of the disease are often misdiagnosed or overlooked [30, 47, 48], which is problematic as studies have shown that treatment success is improved in the early stages of the disease [48, 49]. This problem persists when using ELISA, as it has been shown that early infection stages don’t give a strong immune response against DD-associated *Treponema* spp. [30, 31]. The importance of an objective-based methodology to diagnose DD cannot be overstated. Moreover, having an efficient monitoring system is crucial. Diagnosing the disease in its early stages not only increases treatment success but also enhances the likelihood of a fast transition from an acute to a healing lesion [48, 49].

In Sweden, the recommended treatment for DD is by directly applying bandages containing salicylic acid to the infection site. After three to five days, the bandages are removed. The cow should be symptom-free after just a few days, and the infection site should be completely healed within a week [50, 51]. In other countries, DD is more commonly treated by having the animals walk through footbaths containing antibacterial substances such as copper sulfate, formaldehyde or antibiotics [12, 52]. The major disadvantage of using footbaths for DD treatment is the risk of spreading disease to other animals in the herd; when incorrectly used, the concentration of antimicrobial substances decreases, and footbaths become contaminated by pathogens, feces, and mud. In addition, footbaths containing antibiotics pose a risk for the spread of antimicrobial resistance [53, 54]. This procedure could be avoided with proper monitoring of DD and using a reliable diagnostic method that could diagnose DD at the herd and individual animal levels. A proper diagnostic method would make it easier to effectively treat individual animals at the early stages of the disease, thereby increasing chances of treatment success. In addition, an ELISA with the capacity to detect DD in the herd by analyzing bulk tank milk would be a useful and effective tool for disease monitoring on a population level, as well as for identifying trends in DD development such as changes in prevalence, geographical distribution, and herd type, which could be of immense value in developing disease control strategies.

In addition to diagnostic methods, further research on the etiology of DD is needed. Infection with DD is thought to have a polymicrobial origin, where multiple species play a role in establishing infection [19, 20]. The gut has been shown to be a reservoir for pathogenic bacteria, and contamination of barn floors by these bacteria has been suggested to play a part in disease transmission [12, 55]. Although many species involved in DD infection have been identified, the characteristics of these bacterial species make them difficult to detect by conventional culture methods [26]. This creates further challenges in truly understanding the progression of the disease and in elucidating which species are important at which stages.

It would be particularly beneficial to repeat this study, with an improved ELISA including additional pathogen specific antigens to analyze how the prevalence of DD has changed over time, as well as to compare the current and past status of individual herds and assess the functionality of the bulk tank milk analysis described herein.

## Conclusion

In conclusion, this study found the apparent prevalence of DD in Swedish dairy herds to be 11.9% assessed by presence of *T. phagedenis* antibodies in bulk tank milk, with the highest occurrence in the southern part of Sweden, where almost one-third of the herds tested positive for the presence of *T. phagedenis* antibodies. Based on available hoof trimming data, we suspect that our results underestimate the true prevalence of DD.

## Data Availability

All data generated or analyzed in this study are included in the article, and raw data can be acquired upon request from the corresponding author.

## Abbreviations

DD: Digital Dermatitis
ELISA: Enzyme-Linked Immunosorbent Assay
PP: Percent Positive
PBST: Phosphate-buffered saline containing 0.5% Tween-20

## References

[1] Cheli R, Mortellaro C. La dermatite digitale del bovino. In: Proceedings of the 8th International Conference on Diseases of Cattle, Milan, Italy. 1974:208–13.

[2] Evans NJ, Murray RD, Carter SD (2016). Bovine digital dermatitis: Current concepts from laboratory to farm. Vet J.

[3] Döpfer D, Koopmans A, Meijer FA, Szakall I, Schukken YH, Klee W (1997). Histological and bacteriological evaluation of digital dermatitis in cattle, with special reference to spirochaetes and *Campylobacter **faecalis*. Vet Rec.

[4] Berry SL, Read DH, Famula TR, Mongini A, Döpfer D (2012). Long-term observations on the dynamics of bovine digital dermatitis lesions on a California dairy after topical treatment with lincomycin HCl. Vet J.

[5] Marti S, Jelinski MD, Janzen ED, Jelinski MJ, Dorin CL, Orsel K (2021). A prospective longitudinal study of risk factors associated with cattle lameness in southern Alberta feedlots. Can J Anim Sci.

[6] Preziuso S, LópezSández CM, Corlevic AT, Beggs DS (2022). Host Factors Impacting the Development and Transmission of Bovine Digital Dermatitis. Ruminants.

[7] Dolecheck KA, Dwyer RM, Overton MW, Bewley JM (2018). A survey of United States dairy hoof care professionals on costs associated with treatment of foot disorders. J Dairy Sci.

[8] Holzhauer M, Hardenberg C, Bartels CJ, Frankena K (2006). Herd- and cow-level prevalence of digital dermatitis in the Netherlands and associated risk factors. J Dairy Sci.

[9] Nielsen BH, Thomsen PT, Green LE, Kaler J (2012). A study of the dynamics of digital dermatitis in 742 lactating dairy cows. Prev Vet Med.

[10] Capion N, Thamsborg SM, Enevoldsen C (2008). Prevalence of foot lesions in Danish Holstein cows. Vet Rec.

[11] Holmøy IH, Ahlén L, Frössling J, Sølverød L, Holzhauer M, Nødtvedt A, Fjeldaas T (2021). Evaluation of test characteristics of 2 ELISA tests applied to bulk tank milk and claw-trimming records for herd-level diagnosis of bovine digital dermatitis using latent class analysis. J Dairy Sci..

[12] Palmer MA, O'Connell NE (2015). Digital Dermatitis in Dairy Cows: A Review of Risk Factors and Potential Sources of Between-Animal Variation in Susceptibility. Animals (Basel).

[13] Frankena K, Somers JG, Schouten WG, van Stek JV, Metz JH, Stassen EN, Graat EA (2009). The effect of digital lesions and floor type on locomotion score in Dutch dairy cows. Prev Vet Med.

[14] Hassall SA, Ward WR, Murray RD (1993). Effects of lameness on the behaviour of cows during the summer. Vet Rec.

[15] Juarez ST, Robinson PH, DePeters EJ, Price EO (2003). Impact of lameness on behavior and productivity of lactating Holstein cows. Appl Anim Behav Sci.

[16] Pavlenko A, Bergsten C, Ekesbo I, Kaart T, Aland A, Lidfors L (2011). Influence of digital dermatitis and sole ulcer on dairy cow behaviour and milk production. Animal.

[17] Gomez A, Cook NB, Socha MT, Döpfer D (2015). First-lactation performance in cows affected by digital dermatitis during the rearing period. J Dairy Sci.

[18] Nielsen MW, Strube ML, Isbrand A, Al-Medrasi WD, Boye M, Jensen TK, Klitgaard K (2016). Potential bacterial core species associated with digital dermatitis in cattle herds identified by molecular profiling of interdigital skin samples. Vet Microbiol.

[19] Caddey B, De Buck J (2021). Meta-Analysis of Bovine Digital Dermatitis Microbiota Reveals Distinct Microbial Community Structures Associated With Lesions. Front Cell Infect Microbiol.

[20] Krull AC, Shearer JK, Gorden PJ, Cooper VL, Phillips GJ, Plummer PJ (2014). Deep sequencing analysis reveals temporal microbiota changes associated with development of bovine digital dermatitis. Infect Immun.

[21] Evans NJ, Brown JM, Demirkan I, Murray RD, Vink WD, Blowey RW (2008). Three unique groups of spirochetes isolated from digital dermatitis lesions in UK cattle. Vet Microbiol.

[22] Trott DJ, Moeller MR, Zuerner RL, Goff JP, Waters WR, Alt DP (2003). Characterization of *Treponema **phagedenis*-like spirochetes isolated from papillomatous digital dermatitis lesions in dairy cattle. J Clin Microbiol.

[23] Evans NJ, Brown JM, Demirkan I, Murray RD, Birtles RJ, Hart CA, Carter SD (2009). Treponema pedis sp. nov., a spirochaete isolated from bovine digital dermatitis lesions. Int J Syst Evol Microbiol..

[24] Oren A, Garrity GM. Valid publication of the names of forty-two phyla of prokaryotes. Int J Syst Evol Microbiol. 2021;71(10):005056. 10.1099/ijsem.0.005056 34694987

[25] Paster BJ, Dewhirst FE. The Phylogenetic Diversity of the Genus *Treponema*. In: Lukehart JDRaSA, editor. U.K: Caister Academic Press; 2006. p. 9–18.

[26] Arrazuria R, Caddey B, Cobo ER, Barkema HW, De Buck J (2021). Effects of different culture media on growth of *Treponema* spp. isolated from digital dermatitis. Anaerobe..

[27] Zinicola M, Lima F, Lima S, Machado V, Gomez M, Döpfer D (2015). Altered Microbiomes in Bovine Digital Dermatitis Lesions, and the Gut as a Pathogen Reservoir. PLoS ONE.

[28] Egger-Danner C, Nielsen P, Fiedler A, Müller K, Fjeldaas T, Döpfer D, et al. ICAR Claw Health Atlas. 2 ed. Experts IWGoFTIWaICH, editor: ICAR, Via Savoia 78, Scala A, Int. 3, 00191, Rome; 2020.

[29] Vink WD, Jones G, Johnson WO, Brown J, Demirkan I, Carter SD, French NP (2009). Diagnostic assessment without cut-offs: application of serology for the modelling of bovine digital dermatitis infection. Prev Vet Med.

[30] Afonso JS, Oikonomou G, Carter S, Clough HE, Griffiths BE, Rushton J (2021). Diagnosis of Bovine Digital Dermatitis: Exploring the Usefulness of Indirect ELISA. Front Vet Sci.

[31] Gomez A, Anklam KS, Cook NB, Rieman J, Dunbar KA, Cooley KE (2014). Immune response against Treponema spp. and ELISA detection of digital dermatitis. J Dairy Sci..

[32] Holzhauer M, Mars J, Holstege M, van der Heijden H (2023). An In-House ELISA for *Treponema* Antibodies in Bulk Milk as Part of a Monitoring Tool for Claw Health in Dairy Herds. Vet Sci..

[33] Murray RD, Downham DY, Demirkan I, Carter SD (2002). Some relationships between spirochaete infections and digital dermatitis in four UK dairy herds. Res Vet Sci.

[34] Demirkan I, Walker RL, Murray RD, Blowey RW, Carter SD (1999). Serological evidence of spirochaetal infections associated with digital dermatitis in dairy cattle. Vet J.

[35] Walker RL, Read DH, Loretz KJ, Hird DW, Berry SL (1997). Humoral response of dairy cattle to spirochetes isolated from papillomatous digital dermatitis lesions. Am J Vet Res.

[36] Frössling J, Rosander A, Björkman C, Näslund K, Pringle M (2018). Detection of Treponema phagedenis-like antibodies in serum and bulk milk from cows with and without digital dermatitis J Vet Diagn Invest.

[37] Aubineau T, Relun A, Gentin B, Guatteo R (2021). Short communication: Informative value of an ELISA applied to bulk tank milk to assess within-herd prevalence of digital dermatitis in dairy herds. J Dairy Sci.

[38] Sergeant E. 2018. Epitools Epidemiological Calculators. Available from: https://epitools.ausvet.com.au/2018. Date accessed: 25^th^ Jan 2024.

[39] Charrad M, Ghazzali N, Boiteau V, Niknafs A (2014). Nbclust: An R Package for Determining the Relevant Number of Clusters in a Data Set. J Stat Softw.

[40] Ahlén L, Fjeldaas T. Digital dermatitis and lameness – an evaluation of locomotion scoring as a tool to detect and control the disease. In: Proceedings of the 20th International Symposium and 12th International Conference on Lameness in Ruminants 2019, Tokyo, Japan.

[41] Barkema HW, von Keyserlingk MA, Kastelic JP, Lam TJ, Luby C, Roy JP (2015). Invited review: Changes in the dairy industry affecting dairy cattle health and welfare. J Dairy Sci.

[42] Nielsen SS, Alvarez J, Bicout DJ, Calistri P, Canali E, Drewe JA (2023). Welfare of dairy cows. EFSA J.

[43] Växa. Health statistics of dairy cattle in Sweden 2019–2020: Växa; 2020 Available from: https://www.sva.se/media/mjppiu2i/health-statistics-2019-2020-v%C3%A4xa.pdf. Date accessed: 8^th^ Nov 2023.

[44] Hultgren J (2003). Lameness and Udder Health in Swedish Dairy Herds, as Influenced by Housing Changes. Acta Vet Scand..

[45] Växa. Husdjursstatistik 2023: Växa; 2023 [updated 13/6/2023]. Available from: https://vxa.qbank.se/mb/?h=c7a1d64e698d8df91094699ba3ffd110&p=dccda36951e6721097a93eae5c593859&display=feature&s=name&d=desc. Accessed 8 Nov 2023.

[46] Gillespie A, Carter SD, Blowey RW, Evans N (2020). Survival of bovine digital dermatitis treponemes on hoof knife blades and the effects of various disinfectants. Vet Rec.

[47] Krull AC, Shearer JK, Gorden PJ, Scott HM, Plummer PJ (2016). Digital dermatitis: Natural lesion progression and regression in Holstein dairy cattle over 3 years. J Dairy Sci.

[48] Solano L, Barkema HW, Jacobs C, Orsel K (2017). Validation of the M-stage scoring system for digital dermatitis on dairy cows in the milking parlor. J Dairy Sci.

[49] Wilson-Welder JH, Alt DP, Nally JE (2015). The etiology of digital dermatitis in ruminants: recent perspectives. Vet Med (Auckl).

[50] Swedish Veterinary Agency (SVA). Digital dermatit hos nötkreatur 2023 [updated 3/3/2023]. Available from: https://www.sva.se/amnesomraden/djursjukdomar-a-o/digital-dermatit-hos-notkreatur/#VetContentx5. Accessed 8 Nov 2023.

[51] Växa. Handbok för klövar Växa; 2022. Available from: https://vxa.qbank.se/mb/?h=2fbcccc4148b3e19d1d34cb849866527&p=dccda36951e6721097a93eae5c593859. Date accessed: 8^th^ Nov 2023.

[52] Laven RA, Proven MJ (2000). Use of an antibiotic footbath in the treatment of bovine digital dermatitis. Vet Rec.

[53] Shearer JK, Hernandez J (2000). Efficacy of two modified nonantibiotic formulations (Victory) for treatment of papillomatous digital dermatitis in dairy cows. J Dairy Sci.

[54] Relun A, Lehebel A, Bareille N, Guatteo R (2012). Effectiveness of different regimens of a collective topical treatment using a solution of copper and zinc chelates in the cure of digital dermatitis in dairy farms under field conditions. J Dairy Sci.

[55] Llor C, Bjerrum L (2014). Antimicrobial resistance: risk associated with antibiotic overuse and initiatives to reduce the problem. Ther Adv Drug Saf.

